# Discovery of key genes as novel biomarkers specifically associated with HPV-negative cervical cancer

**DOI:** 10.1016/j.omtm.2021.03.026

**Published:** 2021-04-06

**Authors:** Yi Liu, Yichi Xu, Wenxiao Jiang, Huihui Ji, Zhi-wei Wang, Xueqiong Zhu

**Affiliations:** 1 Center of Uterine Cancer Diagnosis & Therapy Research of Zhejiang Province, Department of Obstetrics and Gynecology, the Second Affiliated Hospital of Wenzhou Medical University, Wenzhou, 325027, China; 2 Department of Pathology, Beth Israel Deaconess Medical Center, Harvard Medical School, Boston, MA 02215, USA

**Keywords:** HPV-negative cervical cancer, HPV-positive cervical cancer, MEX3A, TTYH3, prognosis, ETV4, HMGA2, PRAME, therapy

## Abstract

Cervical cancer is a common female malignancy that is mainly caused by human papillomavirus (HPV) infection. However, the incidence of HPV-negative cervical cancer has shown an increasing trend in recent years. Because the mechanism of HPV-negative cervical cancer development is unclear, this study aims to find the pattern of differential gene expression in HPV-negative cervical cancer and verify the underlying potential mechanism. Differentially expressed genes were compared among HPV-positive cervical cancer, HPV-negative cervical cancer, and normal cervical tissues retrieved from TCGA. Subsequently, dysregulated differentially expressed genes specifically existed in HPV-negative cervical cancer tissues and HPV-negative cell lines were validated by qRT-PCR, western blotting, and immunohistochemical staining. We found seventeen highly expressed genes that were particularly associated with HPV-negative cervical cancer from analysis of TCGA database. Among the 17 novel genes, 7 genes (preferentially expressed antigen in melanoma [PRAME], HMGA2, ETS variant 4 [ETV4], MEX3A, TM7SF2, SLC19A1, and tweety-homologs 3 [TTYH3]) displayed significantly elevated expression in HPV-negative cervical cancer cells and HPV-negative cervical cancer tissues. Additionally, higher expression of MEX3A and TTYH3 was associated with a shorter overall survival of patients with HPV-negative cervical cancer. Our study implies that these seven genes are more likely to provide novel insights into the occurrence and progression of HPV-negative cervical cancer.

## Graphical abstract

**Figure:**
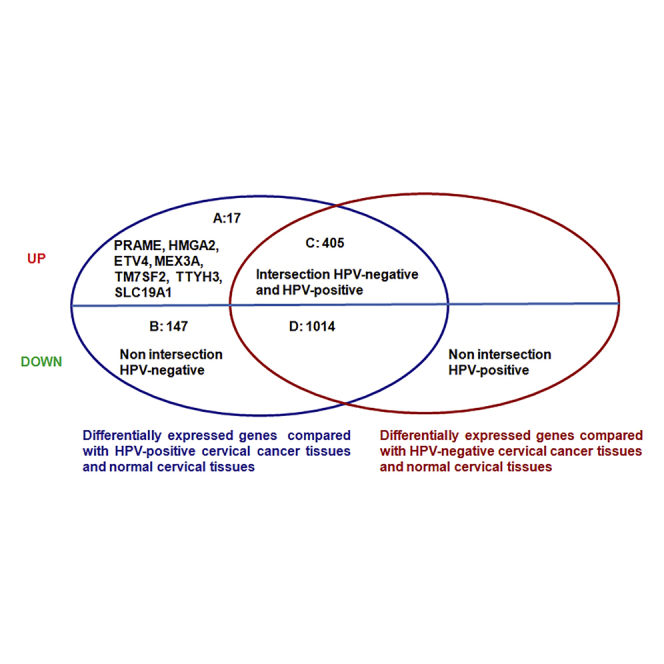

## Teaser

The mechanisms of HPV-negative cervical cancer are largely elusive. In this study, the authors discovered the multiple genes in HPV-negative cervical cancer, including PRAME, HMGA2, ETV4, MEX3A, TM7SF2, SLC19A1, and TTYH3, which could contribute to HPV-negative cervical cancer development.

## Introduction

Cervical cancer is a common female malignancy worldwide, with nearly 600,000 cases and more than 300,000 deaths every year.^1^ The mortality rate in developed countries is lower than in developing countries because of the successful cytological screening and high rate of prophylactic human papillomavirus (HPV) vaccination in developed countries.^1^^,^^2^ An estimated 14,480 patients will be diagnosed and an estimated 4,290 females will die from cervical cancer in the United States in 2021.^3^ Persistent HPV infection, especially high-risk HPV, is considered the principal cause of this disease.^4^ Recently, emerging studies have demonstrated the occurrence of HPV-negative cervical cancers, which may be attributed to the existence of risk factors, such as a weakened immune system, older age, chlamydia infection, and family history other than HPV infection in the occurrence of cervical squamous cell carcinoma.^5^^,^^6^ In addition, compared to patients with HPV-positive cervical cancer, patients with HPV-negative cervical cancer are more likely to skew to older age and present an overall worse outcome, as observed by a shorter survival period, advanced stages, and higher prevalence of lymph node metastases.^6^, ^7^, ^8^

Some previous studies have revealed the different gene expression patterns of HPV-negative and HPV-positive subtypes of cervical cancers, such as Oct4, the long noncoding RNA (lncRNA) PVT1, and lncRNA SRA1,^9^, ^10^, ^11^ implying that various molecular pathways are involved. However, these studies did not systematically analyze and verify specific genes related to the development of HPV-negative cervical cancer. We aim to characterize the differential expression of genes that may constitute novel and comprehensive biomarkers in HPV-negative cervical cancers. In order to achieve this goal, we selected possible HPV-negative-related genes from the Cancer Genome Atlas (TCGA) database, which were further validated by real-time RT-PCR at the mRNA levels and western blotting analysis at the protein-expression levels. Additionally, we also detected the protein expression of these genes by immunohistochemistry (IHC) in HPV-positive cervical cancer tissues compared with HPV-negative cervical cancer tissues and normal control tissues. Identifying novel biomarkers might have the potential to provide novel insights into the specific mechanisms of carcinogenesis in HPV-negative cervical carcinoma.

## Results

### Significantly differentially expressed genes are involved in HPV-negative cervical cancer

**Figure 1:**
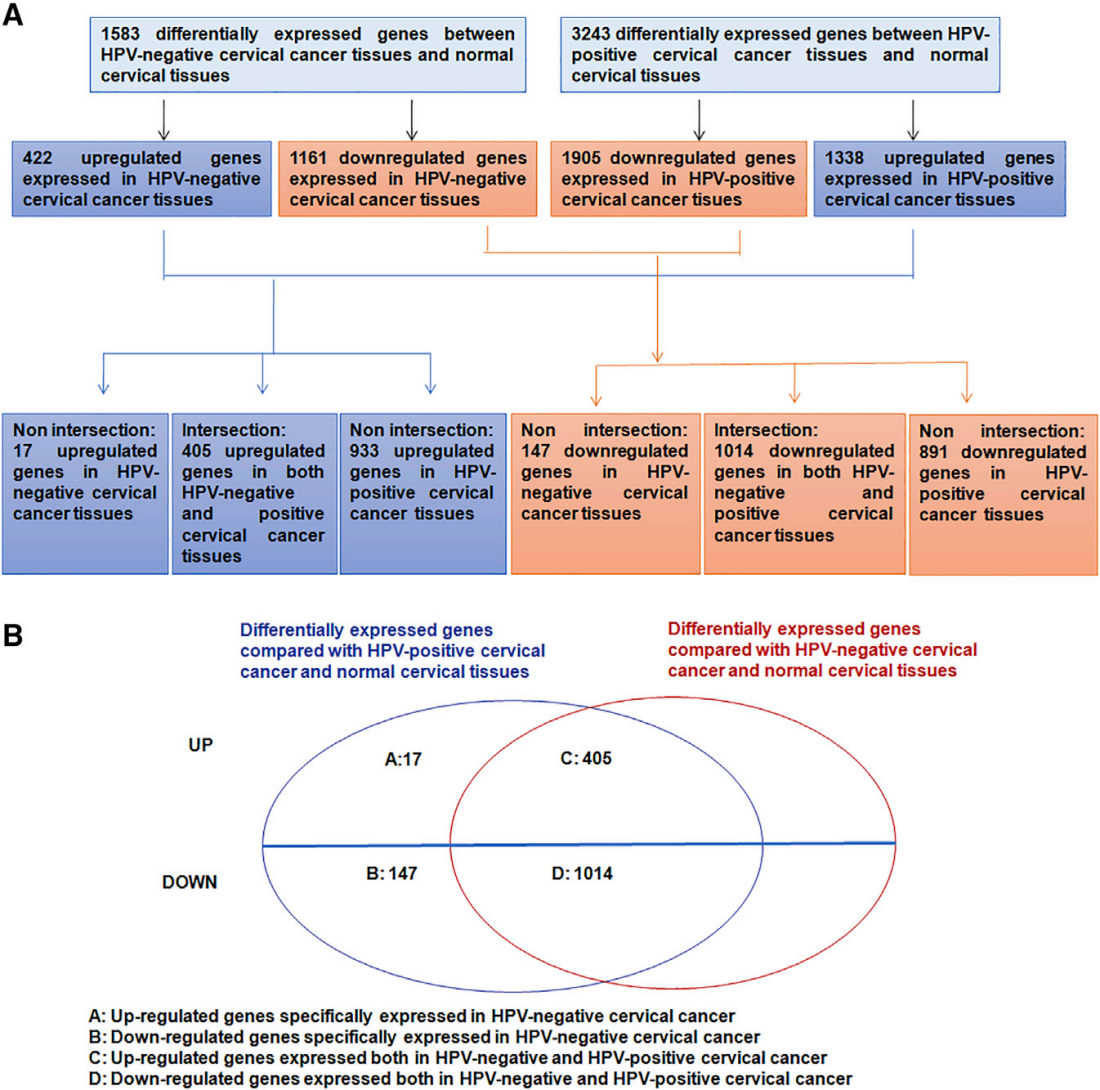
Differentially expressed genes in HPV-negative cervical cancer (A) Workflow of the analysis of differentially expressed genes used in the study. (B) Differentially expressed genes specifically associated with HPV-negative cervical cancer.

In an effort to systemically understand genes related to HPV-negative cervical cancer development (Figure 1A), an analysis of the RNA sequencing (RNA-seq) data identified 1,583 differentially expressed genes between HPV-negative cervical cancer and normal cervical tissues and 3,243 differentially expressed genes between HPV-positive cervical cancer and normal cervical tissues at the cutoff p < 0.01. Among the 1,583 differentially expressed genes identified between HPV-negative cervical cancer and normal cervical tissues, 422 genes were significantly upregulated and 1,161 genes were significantly downregulated. Moreover, among the 3,243 differentially expressed genes identified between HPV-positive cervical cancer and normal cervical tissues, 1,338 genes were significantly upregulated and 1,905 genes were significantly downregulated (Figure 1A). Then, we compared the significantly upregulated and downregulated differentially expressed genes between HPV-negative and HPV-positive samples. As shown in Figure 1B, 17 upregulated genes specifically expressed in HPV-negative cervical cancer were identified, including preferentially expressed antigen in melanoma (PRAME), HMGA2, MT1G, SBK1, E26 transformation specificity (ETS) variant 4 (ETV4), SLC7A5, MEX3A, PLS1, TM7SF2, PYCR1, SLC19A1, RHPN1, CHD7, tweety-homologs 3 (TTYH3), HSPH1, SLC25A13, and NIPSNAP1. Furthermore, 147 downregulated genes were specifically expressed in HPV-negative cervical cancer, and 405 upregulated and 1,014 downregulated genes were expressed in both HPV-negative and HPV-positive cervical cancer.

### Higher mRNA levels of 17 differentially expressed genes are observed in HPV-negative cervical cancer cells

**Figure 2:**
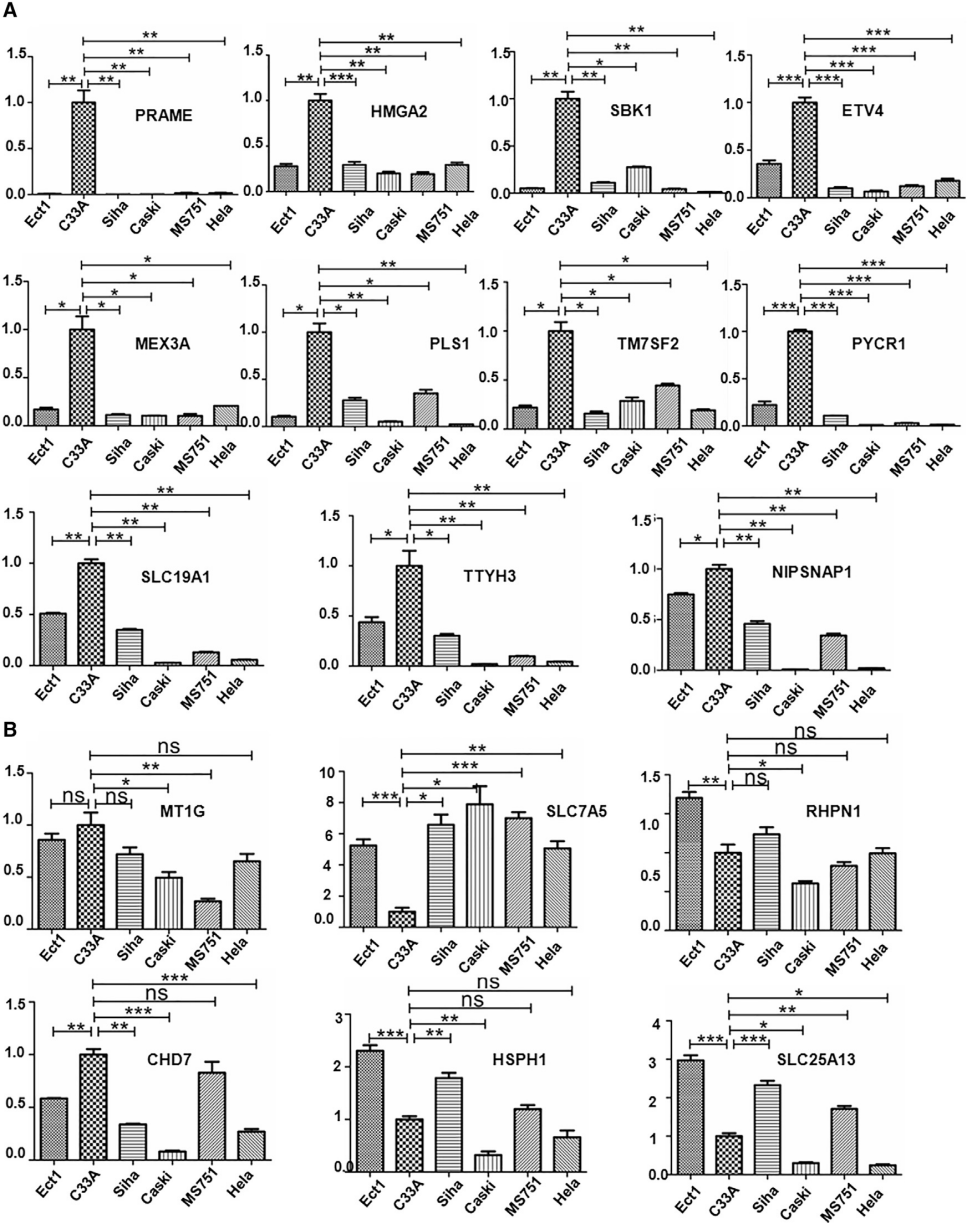
The mRNA expression of seventeen upregulated genes in HPV-negative cervical cancer cells C33A is an HPV-negative cervical cancer cell line. SiHa, Caski, MS751, and HeLa cells are HPV-positive cervical cancer cell lines. Ect1 is a normal cervical squamous cell line. (A and B) The mRNA expression of 17 upregulated genes, including PRAME, HMGA2, SBK1, ETV4, MEX3A, PLS1, TM7SF2, NIPSNAP1, PYCR1, SLC19A1, TTYH3 (A), MT1G, SLC7A5, RHPN1, CHD7, HSPH1, and SLC25A13 (B) was detected in different cells. ∗p < 0.05, ∗∗p < 0.01, ∗∗∗p < 0.001, and ns, not significant. Data are presented as the means ± SD from triplicate experiments.

qRT-PCR assays were used to detect the mRNA expression of these 17 genes in HPV-negative cervical cancer cells (C33A) and HPV-positive cervical cancer cells (SiHa, Caski, MS751, and HeLa) and normal cervical squamous cells (Ect1) to further examine the 17 differentially expressed genes that were specifically involved in HPV-negative cervical cancer. Eleven genes showed significantly elevated mRNA expression in C33A cells compared to SiHa, Caski, MS751, HeLa, and Ect1 cells. The 11 significantly expressed genes were PRAME, HMGA2, SBK1, ETV4, MEX3A, PLS1, TM7SF2, PYCR1, SLC19A1, TTYH3, and NIPSNAP1 (Figure 2A). However, the mRNA levels of the other six genes were not expressed at significantly higher levels in HPV-negative cells than in other cells (Figure 2B).

### Higher protein levels of expressed genes are detected in HPV-negative cervical cancer cells

**Figure 3:**
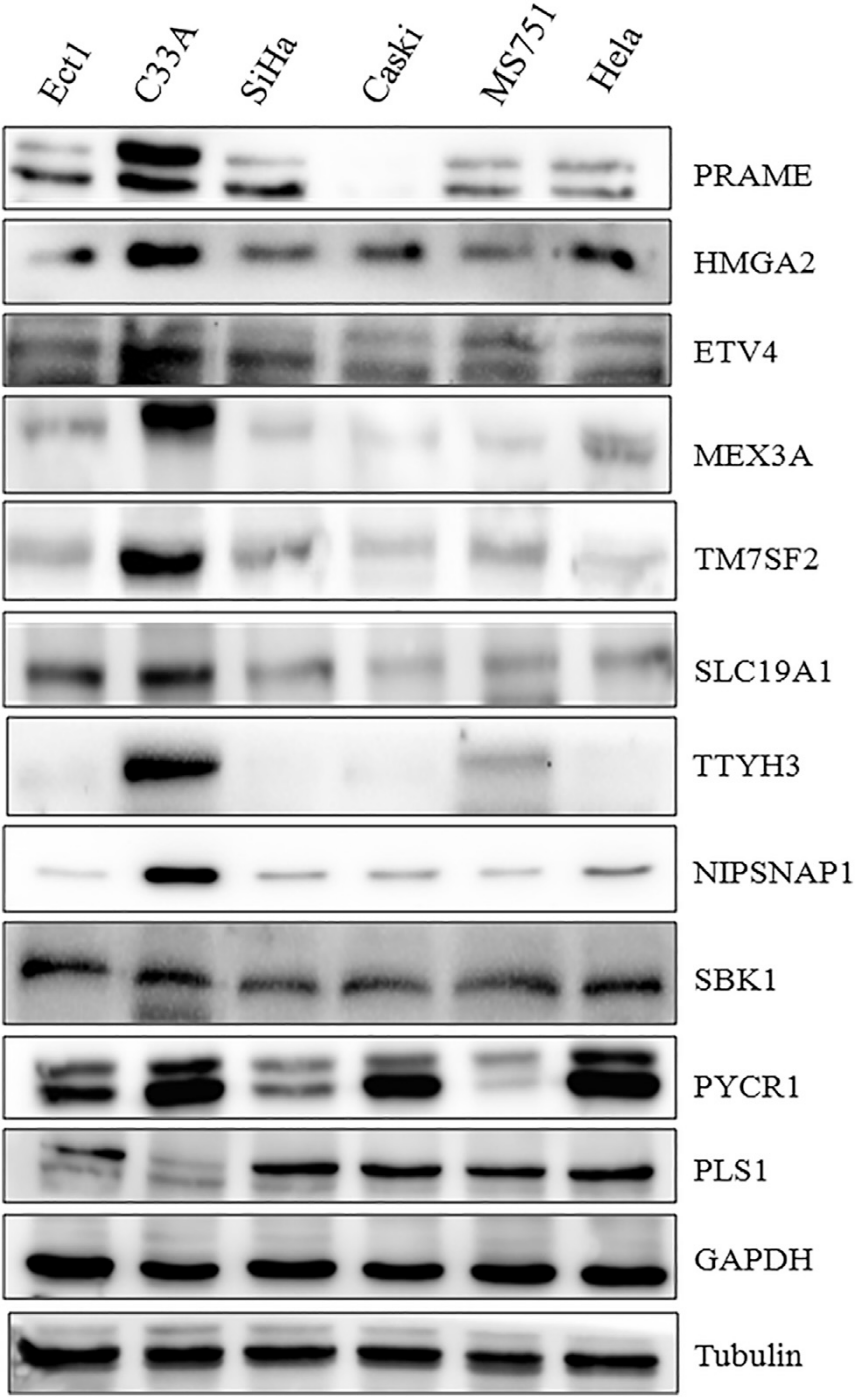
The protein levels of eleven upregulated genes in HPV-negative cervical cancer cells C33A is an HPV-negative cervical cancer cell line. SiHa, Caski, MS751, and HeLa cells are HPV-positive cervical cancer cell lines. Ect1 is a normal cervical squamous cell line. Levels of the PRAME, HMGA2, ETV4, MEX3A, TM7SF2, SLC19A1, TTYH3, NIPSNAP1, SBK1, PYCR1, and PLS1 proteins in different cells were determined by western blotting.

We assessed the protein levels of these 11 genes (PRAME, HMGA2, SBK1, ETV4, MEX3A, PLS1, TM7SF2, PYCR1, SLC19A1, TTYH3, and NIPSNAP1) in HPV-negative and HPV-positive cervical cancer cell lines using western blotting. The protein levels of eight specific genes, PRAME, HMGA2, ETV4, MEX3A, TM7SF2, SLC19A1, TTYH3, and NIPSNAP1, were higher in C33A cells than in SiHa, Caski, MS751, HeLa, and Ect1 cells (Figure 3). However, no remarkable differences in SBK1 levels were observed in all cell lines. Interestingly, PYCR1 showed a higher expression in C33A cells than in SiHa, Caski, MS751, and Ect1 cells, but the differences between C33A and HeLa cells were not significant. Intriguingly, in contrast to the results obtained at the mRNA level, lower expression of the PLS1 protein was detected in C33A cells than in any other cell line (Figure 3).

### Upregulation of proteins is observed in HPV-negative cervical cancer tissues

**Figure 4:**
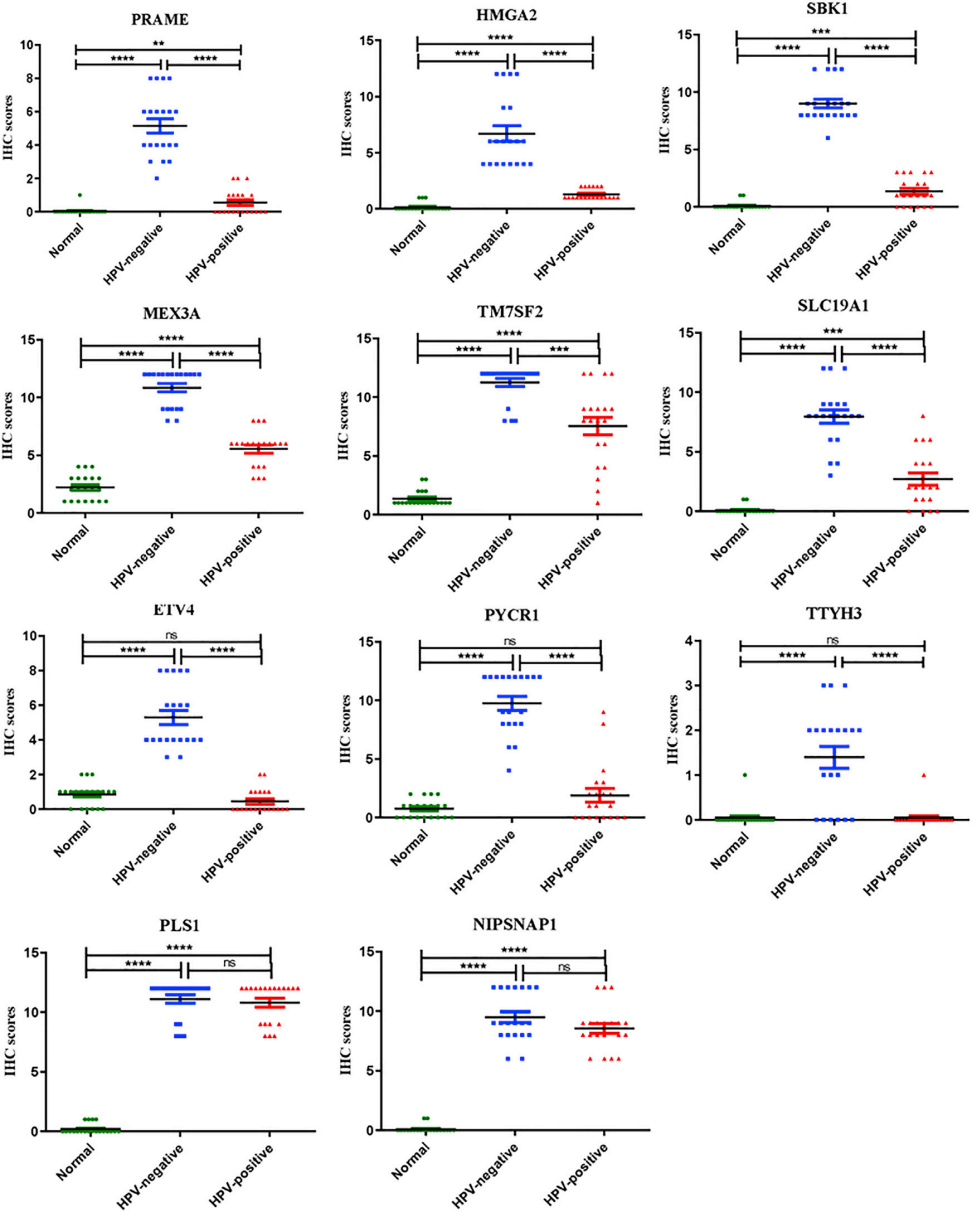
The protein levels of eleven upregulated genes in HPV-negative cervical cancer tissues The protein expression level was determined by calculating immunohistochemical staining score. Immunohistochemical staining scores for 11 proteins, PRAME, HMGA2, SBK1, MEX3A, TM7SF2, SLC19A1, ETV4, PYCR1, TTYH3, PLS1, and NIPSNAP1, in HPV-negative cervical cancer tissues, HPV-positive cervical cancer tissues, and normal cervical tissues are shown. ∗∗p < 0.01, ∗∗∗p < 0.001, and ∗∗∗∗p < 0.0001. Data are presented as the means ± SD from triplicate experiments.

**Figure 5:**
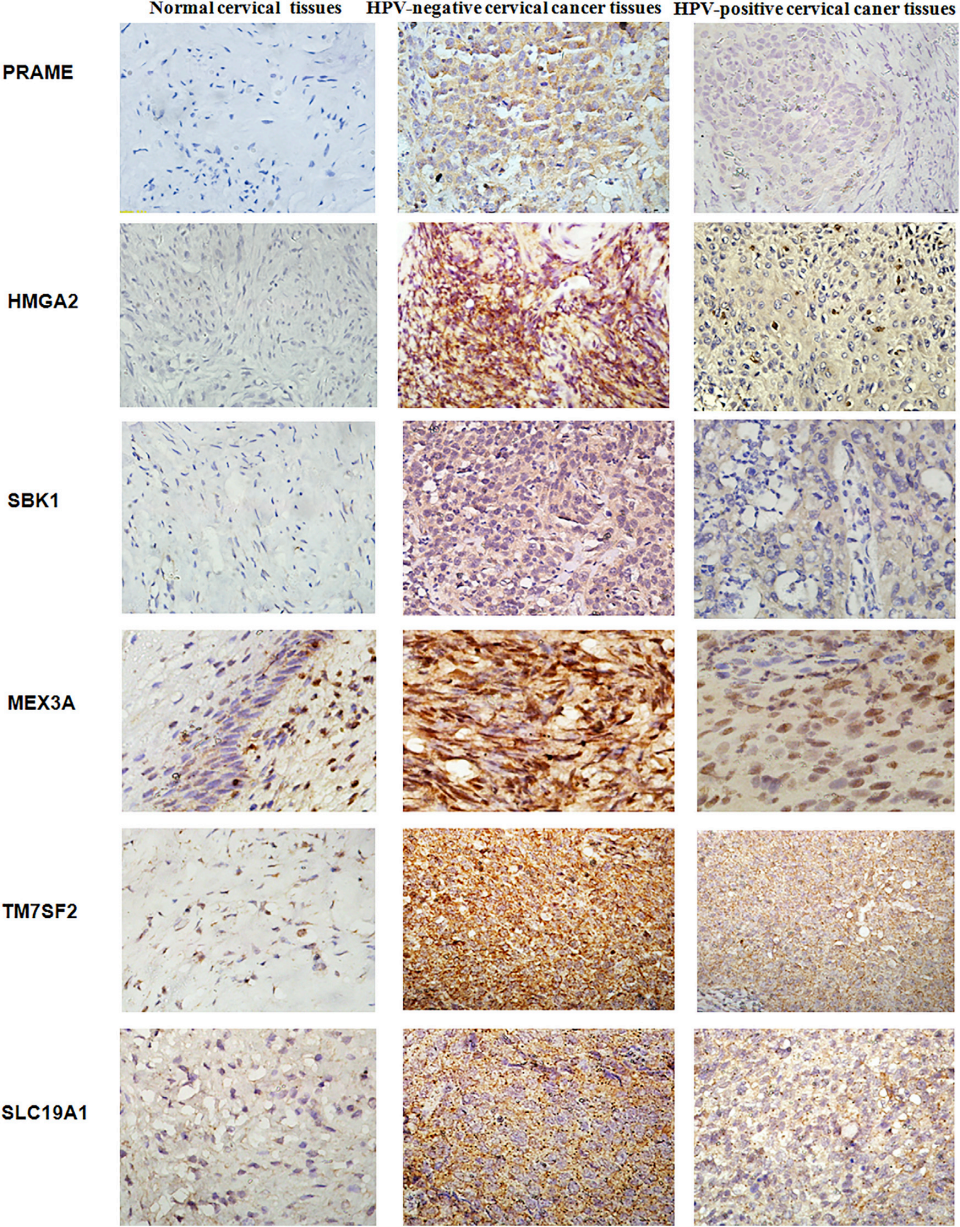
Representative images of immunohistochemical staining for several genes The images of PRAME, HMGA2, SBK1, MEX3A, TM7SF2, and SLC19A1 were presented in HPV-negative cervical cancer tissues, HPV-positive cervical cancer tissues, and normal cervical tissues.

**Figure 6:**
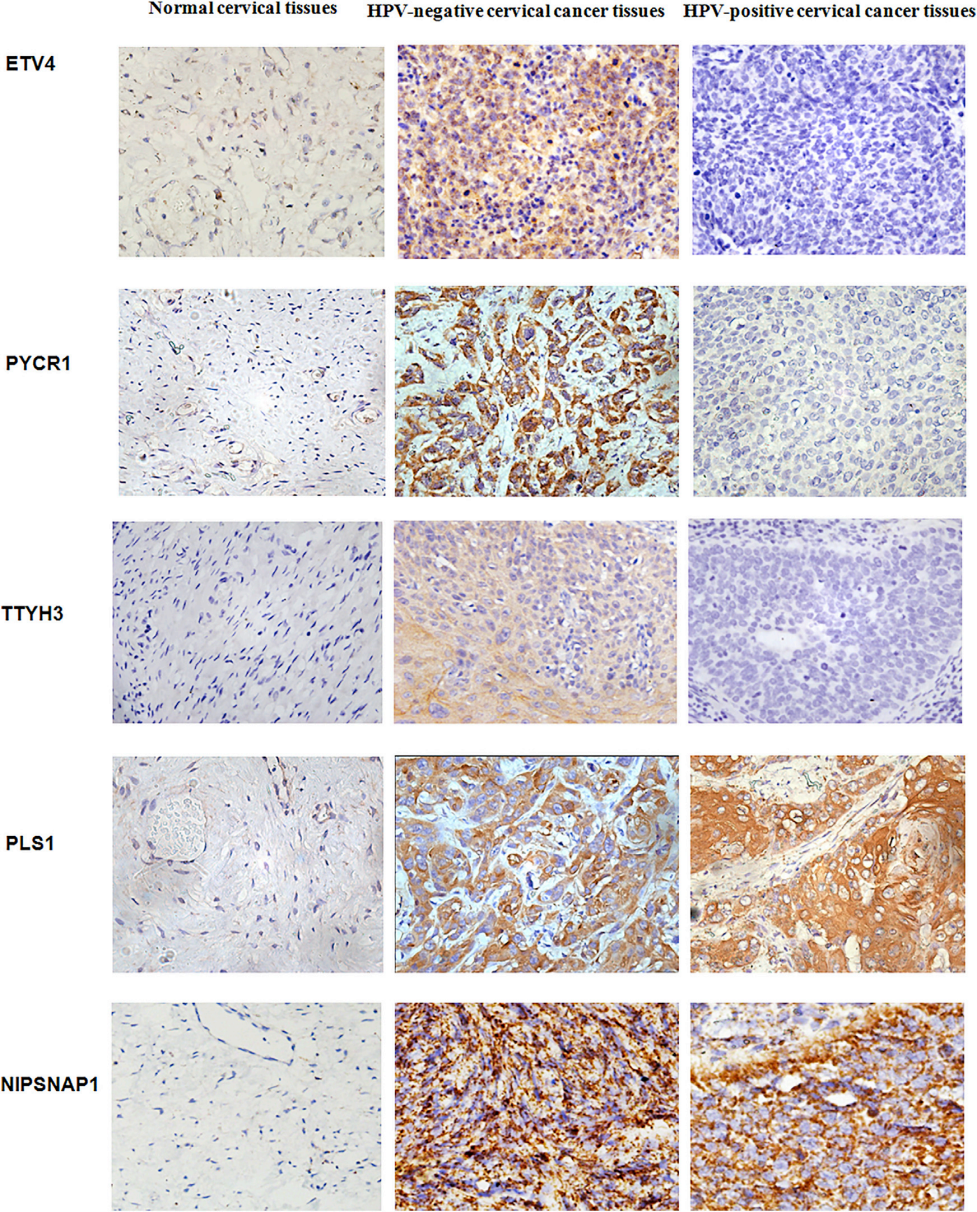
Representative image of immunohistochemical staining for several genes The images of ETV4, PYCR1, TTYH3, PLS1, and NIPSNAP1 were presented in HPV-negative cervical cancer tissues, HPV-positive cervical cancer tissues, and normal cervical tissues.

We conducted IHC staining to further validate differences in the protein expression of 11 specific genes, PRAME, HMGA2, SBK1, ETV4, MEX3A, PLS1, TM7SF2, PYCR1, SLC19A1, TTYH3, and NIPSNAP1, in HPV-negative cervical cancer in this study. The mean IHC scores for these 11 genes in HPV-negative cervical cancer, HPV-positive cervical cancer, and normal cervical tissues were shown in Figure 4. Higher expression of PRAME, HMGA2, SBK1, MEX3A, TM7SF2, and SLC19A1 was detected in HPV-negative and HPV-positive cervical cancer tissues than in normal cervical tissues, and these 6 proteins were also expressed at higher levels in HPV-negative cervical cancer tissues than HPV-positive cervical cancer tissues (Figure 5). Furthermore, significantly elevated levels of the ETV4, PYCR1, and TTYH3 proteins were observed in HPV-negative cervical cancer tissues, but these proteins were rarely expressed in HPV-positive cervical cancer and normal cervical tissues (Figure 6). Moreover, the expression levels of PLS1 and NIPSNAP1 were higher in patients with HPV-positive and HPV-negative cervical cancer than in the normal group, but no significant differences were observed between HPV-positive patients and HPV-negative patients (Figure 6). Therefore, 9 genes were specifically expressed in HPV-negative cervical cancer, including PRAME, HMGA2, SBK1, MEX3A, TM7SF2, SLC19A1, ETV4, PYCR1, and TTYH3, compared to HPV-positive cervical cancer and normal cervical tissues.

### Prognostic value of upregulated differentially expressed genes in patients with HPV-negative cervical cancer, patients with HPV-positive, and all patients with cervical cancer

**Figure 7:**
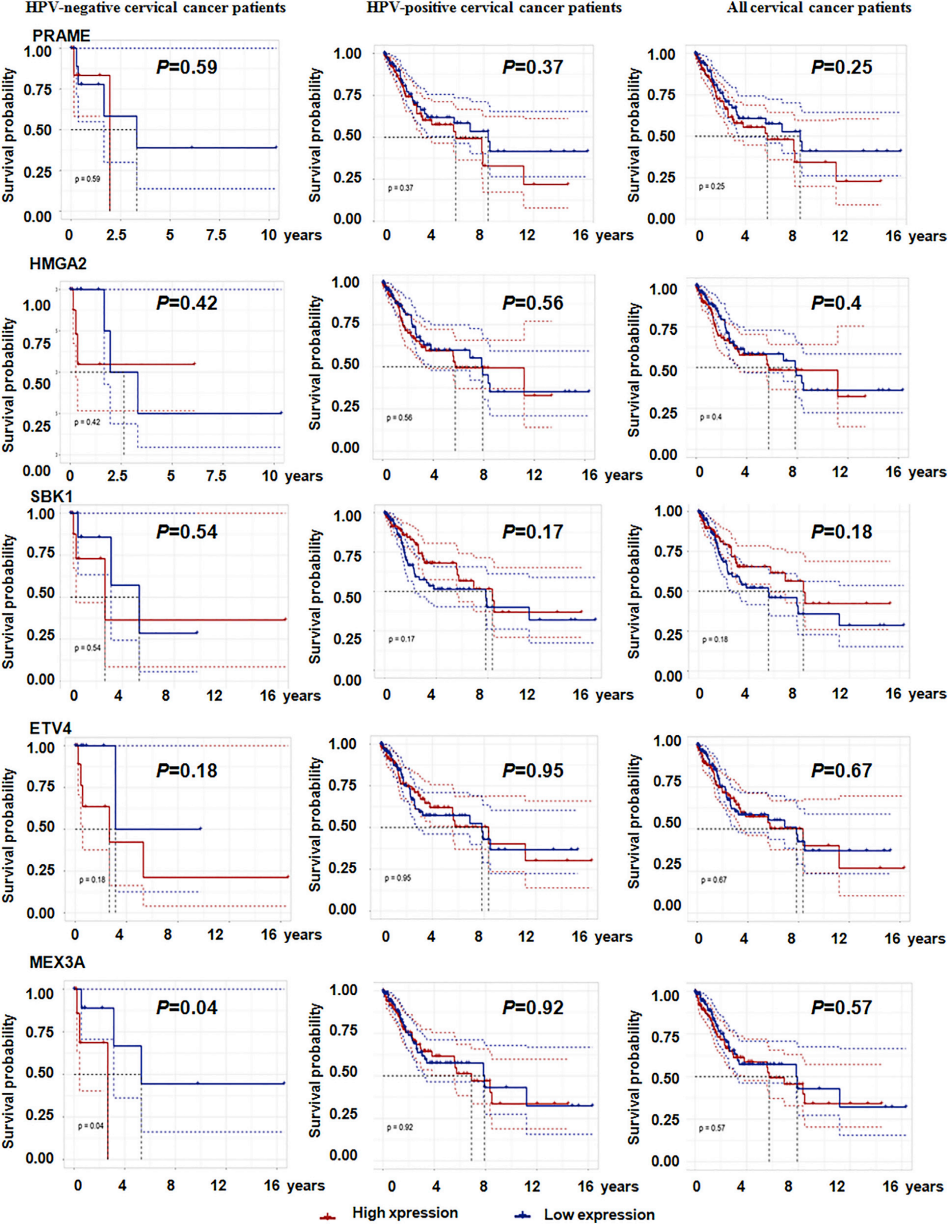
The correlations between five genes and survival rates are shown in cervical cancer patients Analysis of the correlations between PRAME, HMGA2, SBK1, ETV4, and MEX3A expression and survival rates in patients with HPV-negative cervical cancer (22 samples), patients with HPV-positive cervical cancer (281 samples), and all patients with cervical cancer (304 samples). All samples information was obtained from the TCGA.

**Figure 8:**
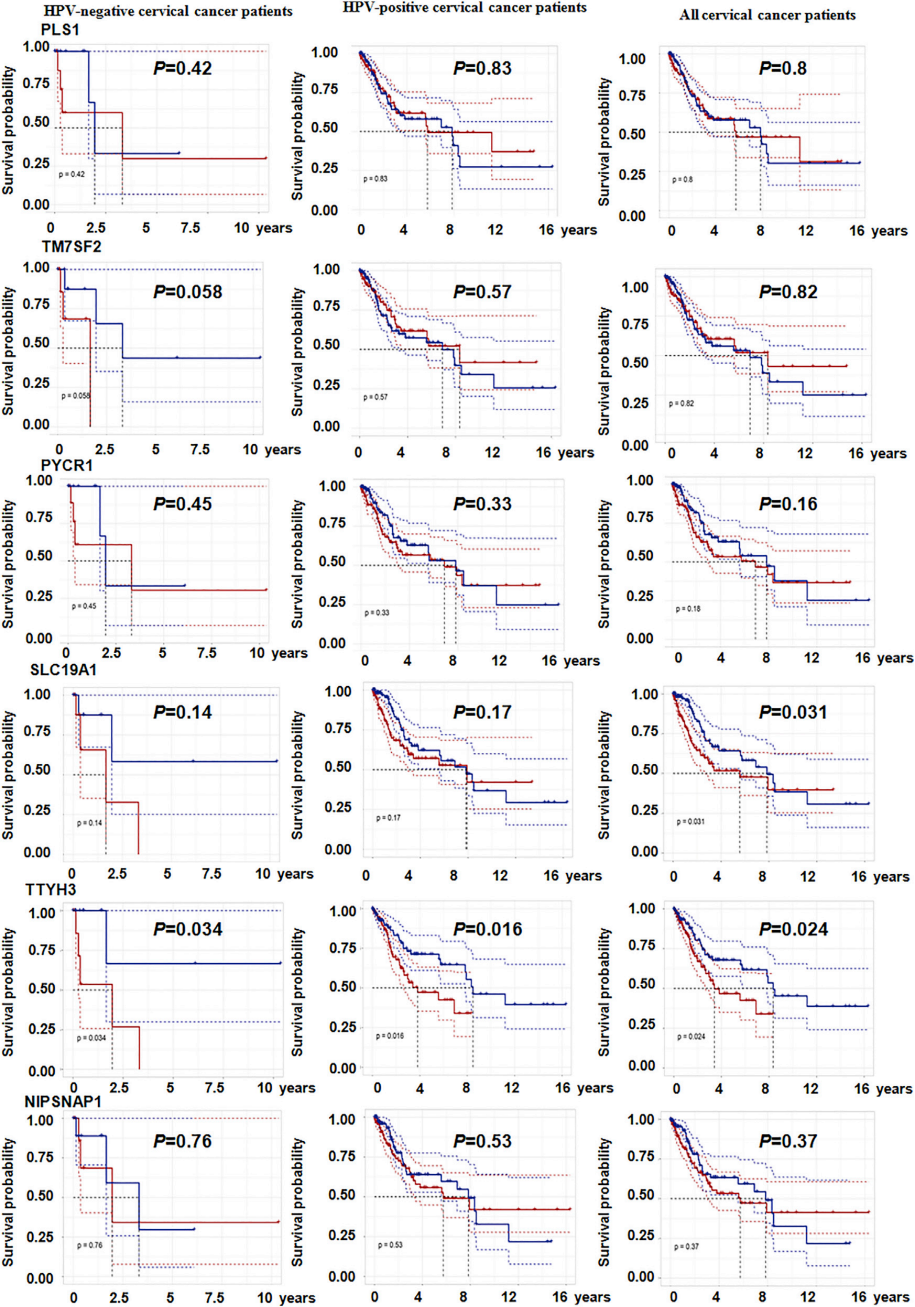
The associations between six genes and survival rates are shown in cervical cancer patients Analysis of the correlation between PLS1, TM7SF2, PYCR1, SLC19A1, TTYH3, and NIPSNAP1 expression and survival rates in patients with HPV-negative cervical cancer (22 samples), patients with HPV-positive cervical cancer (281 samples), and all patients with cervical cancer (304 samples). All samples information was obtained from the TCGA.

Finally, the prognostic value of the 11 upregulated genes (PRAME, HMGA2, SBK1, ETV4, MEX3A, PLS1, TM7SF2, PYCR1, SLC19A1, TTYH3, and NIPSNAP1) specifically associated with HPV-negative cervical cancer was assessed by performing a Kaplan-Meier analysis of patients with HPV-negative cervical cancer, patients with HPV-positive cervical cancer, and all patients with cervical cancer. Higher expression of MEX3A was associated with shorter overall survival only in patients with HPV-negative cervical cancer (p = 0.04) but was not associated with the survival of patients with HPV-positive cervical cancer and all patients with cervical cancer (Figure 7). Additionally, elevated TTYH3 expression was correlated with shorter overall survival in patients with HPV-negative cervical cancer (p = 0.034), patients with HPV-positive cervical cancer (p = 0.016), and all patients with cervical cancer (p = 0.024). Moreover, a high level of SLC19A1 correlated with a poor prognosis for all patients with cervical cancer (p = 0.031). Nevertheless, other genes showed no association with the prognosis of cervical cancer (Figure 8).

## Discussion

HPV DNA detection for cervical cancer screening is increasingly used as an early diagnostic guideline to prevent the progression of cervical cancer. Nevertheless, an ongoing need exists for studies investigating the molecular mechanisms related to prevent the development of cervical cancer. Recently, some lncRNAs were reported to be downregulated in HPV-negative cervical cancer. For example, the lncRNA snaR was downregulated in patients with HPV-negative cervical squamous cell carcinoma.^12^ Additionally, the expression of the lncRNA neighboring enhancer of FOXA2 (NEF) was significantly downregulated in tissues and serum of patients with HPV-negative cervical squamous cell carcinoma compared to HPV-positive patients and healthy controls, but significant differences were not observed between healthy controls and HPV-positive patients.^13^ Remarkably, Liu et al.^14^ detected the downregulation of SRA1 in patients with HPV-negative cervical squamous cell carcinoma, but not HPV-positive patients. However, upregulated genes involved in HPV-negative cervical cancer are rarely reported. A recent study observed significantly higher expression of the lncRNA PVT1 in patients with HPV-positive cervical cancer and patients with HPV-negative cervical cancer than in the control group. However, no significant differences in PVT1 levels were observed between HPV-positive patients and HPV-negative patients.^11^

In the present study, we first compared changes in gene expression between HPV-negative cervical cancer and normal cervical tissues, as well as between HPV-positive cervical cancer and normal cervical tissues to identify differentially expressed genes. Then, we further compared the genes that were expressed at significantly higher levels in HPV-negative cervical cancer tissues than in HPV-positive cervical cancer tissues to discover genes that were specifically expressed at higher levels in HPV-negative cervical cancer. Ultimately, 17 genes specifically upregulated in HPV-negative cervical cancer were found. A subsequent multiplatform integrative analysis revealed that 11 genes, PRAME, HMGA2, SBK1, ETV4, MEX3A, PLS1, TM7SF2, PYCR1, SLC19A1, TTYH3, and NIPSNAP1, displayed upregulated mRNA levels, and 8 specific genes, PRAME, HMGA2, ETV4, MEX3A, TM7SF2, SLC19A1, TTYH3, and NIPSNAP1, displayed upregulated protein levels in HPV-negative cervical cancer cells compared to HPV-positive cervical cancer cells and normal cervical squamous cells. Consistent with these findings, PRAME, HMGA2, SBK1, MEX3A, TM7SF2, SLC19A1, ETV4, PYCR1, and TTYH3 showed higher expression in HPV-negative cervical cancer tissues than in HPV-positive and normal cervical tissues. Accordingly, 7 genes, PRAME, HMGA2, ETV4, MEX3A, TM7SF2, SLC19A1, and TTYH3, displayed consistent results in terms of mRNA and protein levels obtained using qPCR, western blotting, and IHC methods, as they showed significantly higher expression in HPV-negative cervical cancer cells and tissues than in HPV-positive cervical cancer and normal cells or tissues, implying that these 7 genes are more likely to play pivotal roles in the development of HPV-negative cervical cancer. Furthermore, high expression of MEX3A and TTYH3 was also related to shorter overall survival of patients with HPV-negative cervical cancer, which confirmed the important prognostic value of MEX3A and TTYH3 expression in patients with HPV-negative cervical cancer.

PRAME, a testis-selective cancer testis antigen (CTA), is associated with cell proliferation, apoptosis, differentiation, and the outcomes and risk of metastasis in human cancers.^15^^,^^16^ PRAME has attracted interest as a candidate target for immunotherapy because it encodes a membrane-bound protein that regulated autologous cytotoxic T-cell-mediated immune responses.^17^ To date, few articles have analyzed PRAME in cervical cancer. One article illustrated that overexpression of PRAME in HeLa cells induced caspase-independent cell death, suggesting that PRAME might function as a tumor suppressor in cervical cancer progression.^18^ Our results revealed low levels of the PRAME mRNA and protein in HeLa cells, other HPV-positive cell lines, and normal cell lines. In contrast, the PRAME mRNA and protein were obviously overexpressed in the HPV-negative C33A cell line and HPV-negative cervical cancer tissues, implying that PRAME may represent a potential biomarker only for HPV-negative cervical cancers.

The architectural transcription factor HMGA2 is a nonhistone DNA-binding factor that tightly binds to adenine and thymine (AT)-rich sequences in the minor groove of the DNA helix.^19^ Jiang et al.^20^ identified that HMGA2 combined with LRP1B and TP63 function as potential biomarkers that are useful for triaging HPV-positive patients, especially CIN2+ patients. Notably, the depletion of HMGA2 counteracted the epithelial-mesenchymal transition and lymph node metastasis by suppressing the attenuated total reflectance (ATR)/Chk1 signaling pathway in cervical cancer.^21^ Consistent with these findings, overexpression of the HMGA2 protein was detected when HPV integrated into flanking regions in cervical cancer.^22^ Two studies validated that HMGA2 is commonly positive in HPV-negative adenocarcinomas of the uterine cervix and corpus^23^ and HPV-negative head and neck squamous cell carcinoma.^24^ However, a previous study did not observe detectable levels of the HMGIC mRNA in three cervical carcinoma cell lines (SW756, SiHa, and HeLa).^25^ In our study, lower HMGA2 expression was detected in the Siha, Caski, MS751, and HeLa cell line than in the HPV-negative cell line C33A at the mRNA and protein levels. Notably, IHC staining also identified a significantly elevated level of HMGA2 in HPV-negative cervical cancer, suggesting that HMGA2 might be a potential biomarker in HPV-negative cervical cancer.

ETV4, a member of the PEA3 subgroup of the ETS transcription factor family,^26^ plays important roles in regulating gene expression during early embryogenesis,^27^ obesity, and diabetes,^28^ as well as oncogenesis,^29^^,^^30^ and its overexpression is involved in the malignant transformation of cells and enhanced tumor cell invasion.^31^^,^^32^ According to a recent study, ETV4 promotes the migration and metastasis of clear-cell renal-cell carcinoma *in vitro* and *in vivo*.^33^ However, research studies on the expression of ETV4 in cervical cancer are rare. Our study illustrated that ETV4 is specifically associated with HPV-negative cervical cancer. Higher levels of the ETV4 were detected in HPV-negative cervical cancer than in HPV-positive cervical cancer and normal cervical tissues, implying that ETV4 might be a special biomarker of HPV-negative cervical cancer.

TM7SF2 is a human sterol reductase with a function that remains mysterious in human cancers.^34^ Bellezza et al.^35^ discovered that the ablation of TM7SF2 resulted in the formation of skin papilloma in mice. The present study is the first to report the association between TM7SF2 and cervical cancer. Based on our results, TM7SF2 was expressed at higher levels in the C33A cell line and HPV-negative tissues than in HPV-positive, normal cell lines and tissues, implying that TM7SF2 may also play a specific role in the development of HPV-negative cervical cancer.

SLC19A1, a folate-organic phosphate antiporter, serves as the major transporter of cyclic dinucleotides, and its depletion and overexpression affect cyclic dinucleotide uptake and functional responses, which has implications for the immunotherapeutic treatment of cancer via cGAS-STING pathway.^36^^,^^37^ In this study, we first identified that SLC19A1 might play an important role, especially in HPV-negative cervical carcinoma. Overexpression of SLC19A1 was detected in C33A cell line, whereas low expression was observed in HPV-positive cell lines, consistent with the results obtained from human tissues, implying SLC19A1 has potential relevance in the carcinogenesis of HPV-negative cervical cancer.

The RNA-binding ubiquitin ligase MEX3A is overexpressed in glioblastoma multiforme specimens, and it targets retinoic acid inducible gene-I (RIG-I) for ubiquitylation and degradation to alter the tumorigenesis of glioblastoma multiforme.^38^ Notably, downregulation of MEX3A inhibited cell growth and migration and promoted apoptosis in pancreatic ductal adenocarcinoma.^39^ Moreover, a higher level of MEX3A was significantly associated with shorter survival of patients with liver cancer.^40^ We found higher expression of MEX3A in HPV-negative cervical carcinoma cell lines and tumor tissues. In addition, upregulation of MEX3A was associated with shorter overall survival only in patients with HPV-negative cervical cancer, implying that MEX3A is not only related to the occurrence of HPV-negative cervical but also to prognosis of HPV-negative cervical cancer.

The TTYH family, chloride-channel-responsive genes, is involved in cell adhesion, cell division, tumorigenesis, and regulation of calcium signaling.^41^ TTYH3 is the third member of the TTYH family harboring chloride channel activities and is known as a large-conductance Ca2^+^-activated chloride channel that acts as the major volume-regulated anion channel (VRAC) in astrocytes, together with TTYH1/2.^42^^,^^43^ A study revealed that the upregulation of the *TTYH3* in gastric cancer was associated with shorter survival.^44^ Nevertheless, little is known about the expression and function of TTYH3 in any other tumors. Our data revealed a potentially important role for TTYH3 in the development of HPV-negative cervical cancer, because higher expression of TTYH3 was observed in HPV-negative cervical cancer cells and tissues. Upregulation of TTYH3 was also associated with shorter survival, suggesting TTYH3 might be a potential prognostic marker for patients with cervical cancer.

In conclusion, to our knowledge, this study is the first to systematically analyze, examine, and disclose differentially expressed genes that are specifically expressed in patients with HPV-negative cervical cancer. Our results firmly identified the significantly increased expression of 8 novel genes at the mRNA and protein levels, including PRAME, HMGA2, ETV4, MEX3A, TM7SF2, SLC19A1, TTYH3, and NIPSNAP1, in HPV-negative cervical carcinoma cell lines compared to HPV-positive cell lines and a normal cell line. Furthermore, significantly higher expression of PRAME, HMGA2, ETV4, MEX3A, TM7SF2, SLC19A1, and TTYH3 was also detected in HPV-negative cervical cancer tissues, implying that these seven genes may serve as new biomarkers for HPV-negative cervical cancer and play pivotal roles in the occurrence and development of HPV-negaive cervical cancer. In addition, MEX3A and TTYH3 correlated with shorter overall survival of patients with HPV-negative cervical cancer, suggesting that the upregulation of MEX3A and TTYH3 were not only associated with the development of HPV-negative cervical cancer but also specifically involved in the prognosis of HPV-negative cervical cancer. In the future, further studies and more analyses will be performed to extensively investigate the effects of changes in the expression of these genes, especially MEX3A and TTYH3, on the biological behavior, chemotherapy sensitivity, related internal mechanisms, and signaling pathways specifically associated with HPV-negative cervical carcinoma to provide new insights into the early screening and effective treatment of HPV-negative cervical carcinoma.

## Materials and methods

### Data resources

Publicly available data from the TCGA database were downloaded on March 15, 2018 from https://www.cancer.gov/about-nci/organization/ccg/research/structural-genomics/tcga. Three hundred five samples of cervical cancer and 3 normal cervical tissues from healthy controls were analyzed; among the cervical cancer tissues, 282 were from patients with a known HPV infection and 23 were from HPV-negative patients.

### Gene expression analysis

The gene expression data were obtained from TCGA public level 2 transcription profiles as raw count values. R packages (edgeR) were applied to the transcription profiles, and differentially expressed genes between HPV-positive cervical cancer and normal cervical tissues and between the HPV-negative cervical cancer and normal cervical samples were analyzed. Compared to the normal cervix, differentially expressed genes in HPV-negative or HPV-positive cervical cancer were divided into four separate groups, including upregulated genes expressed in HPV-negative cervical cancer, downregulated genes expressed in HPV-negative cervical cancer, upregulated genes expressed in HPV-positive cervical cancer, and downregulated genes expressed in HPV-positive cervical cancer. Then, we compared the upregulated genes expressed in HPV-negative and HPV-positive cervical cancer, and two non-intersecting groups and one intersecting group were obtained, which represent upregulated genes expressed specifically in HPV-negative or -positive cervical cancer and upregulated genes expressed in both HPV-negative and HPV-positive cervical cancer, respectively. Using the same method, we compared downregulated genes expressed in HPV-negative and HPV-positive cervical cancer, and two other non-intersecting groups (downregulated genes expressed specifically in HPV-negative cervical cancer and downregulated genes expressed specifically in HPV-positive cervical cancer) and an intersecting group (downregulated genes expressed in both HPV-negative and HPV-positive cervical cancer) were obtained. Finally, upregulated differentially expressed genes specifically detected in HPV-negative cervical cancer were identified.

### Cell lines and cell culture

The normal cervical squamous cell line Ect1 was used, in addition to the human cervical carcinoma cell lines, C33A (HPV-negative) and SiHa, Caski, MS751, and HeLa (HPV-positive). Cells were purchased from the European Collection of Authenticated Cell Cultures and cultured in Dulbecco’s modified Eagle’s medium (DMEM) (Gibco, USA) containing 10% fetal bovine serum at 37°C in an atmosphere of 5% CO_2_ for growth.

### qRT-PCR

TRIzol reagent (Invitrogen; Thermo Fisher Scientific) was used to completely lyse and extract total RNA from HPV-positive, HPV-negative, and normal cells. The total RNA was dissolved in diethyl pyrocarbonate (DEPC)-treated deionized water and quantified with a spectrophotometer at 260 nm (A260). RNA gel electrophoresis was conducted to examine DNA contamination. 1 μg RNA was used for reverse transcription reactions by TransStart Top Green qPCR Supermix (TransGen Biotech). The cDNA templates were synthesized, and qPCR was conducted using the SYBR Green Real-Time PCR Master mix (Applied Biosystems; Thermo Fisher Scientific). The following thermocycling conditions were used: 95°C for 55 s followed by 40 cycles of 95°C for 10 s and 57°C for 30 s. Then, gene expression was determined using a LightCycler 480 II real-time PCR (Roche) instrument and normalized to glyceraldehyde-3-phosphate dehydrogenase (GAPDH) using the 2^−ΔΔCt^ method. All PCR reactions were performed in triplicate. The primers for the tested genes are listed in Table 1.

**Table 1:** The primers of HPV-negative specifically upregulated genes

Type	Gene name	Forward primer (5′->3′)	Reverse primer (5′->3′)
Human	PRAME	CGTGCCTGAGCAACTGAT	TACCCACCTTGGCGAAAT
Human	HMGA2	TGGGGCAGGAACTCAGAAAACT	GCACAGGCAGAGGACAGAGTAGT
Human	MT1G	CCTGGATTTTACGGGTCA	AAAGGGGCATCGGAGAAG
Human	SBK1	CCCGCACATACACGCACTT	CTCCTCGCCATCCCATTCTG
Human	ETV4	CATTTGCCACTCCTTCACATCTCAG	CAGGGGATCATGGTATTCTTGCTTAA
Human	SLC7A5	ACGCCCAGGTGATAGTTCC	CATTATACAGCGGCCTCTTTG
Human	MEX3A	GGCTTGCGTGGCTGTGATG	AGTGACTGCCGCCCTTGTG
Human	PLS1	TGTTCTTCCCAAGTTCCA	TCTATGAGATGATCCGAGTG
Human	TM7SF2	CAGCATGAAGCCAAACCC	TCTGTGAACTGCGACCCG
Human	RHPN1	ACGGCAGCGATGAGGACA	TAAGACCCACCAGAAGCCAGAG
Human	PYCR1	ATCAATCAGGTCCTCTTCCAC	AAGCTGTCAGCGTTTCGG
Human	SLC19A1	CGTCCGAGACAATGAAAGTGA	CATCTGGCTGTGCTATGCG
Human	CHD7	CATGCCTATAAATGGACGA	TGGGATACCAATGGAAGTT
Human	TTYH3	GCGGGAAGCACAGCAAAC	TGTCCTCGGGAATGAACCTC
Human	HSPH1	CCACCATAGATGCCGTAG	AAAATAGGCCGCTTTGTA
Human	SLC25A13	CATAGCGTAGCACTTTCTTA	GTTTGGTCTGGGTTCTGT
Human	NIPSNAP1	CTGAACCTCCTCCTGACC	CCCTTGGAAATCCTCTGT

### Western blotting

The primary antibodies included PRAME, HMGA2, SBK1, ETV4, MEX3A, PLS1, TM7SF2, PYCR1, SLC19A1, TTYH3, NIPSNAP1 GAPDH, and tubulin (the specific information and dilution factor of each antibody are shown in Table 2). Western blotting (WB) analysis was performed as described previously.^45^ Each experiment was repeated three times.

**Table 2:** The primary antibodies of genes in western blotting and IHC

Gene	Type	WB	IHC
PRAME	Abcam, ab219650	1:1,000	1:100
	Biorbyt, orb373396		
HMGA2	Abcam, ab52039	1:1,000	1:50
SBK1	Novus, NBP1-74148	1:1,000	1:100
ETV4	Abcam, ab70425	1:1,000	1:200
MEX3A	Abcam, ab79046	1:1,000	1:100
PLS1	Santa Cruz Biotechnology	1:800	1:250
	Sc-271223		
TM7SF2	Biorbyt, orb4574	1:1,000	1:500
PYCR1	Proteintech, 13108-1-AP	1:1,000	1:200
SLC19A1	Santa Cruz Biotechnology	1:800	1:250
	Sc-390948		
TTYH3	Sigma	1:1,000	1:100
NIPSNAP1	Santa Cruz Biotechnology	1:800	1:250
	Sc-271223		
TUBULIN	Boster, BM145	1:2,000	
GAPDH	Abcam, ab181602	1:2,000	

### Immunohistochemical staining

All tissue samples were obtained from patients with cervical cancer who underwent radical hysterectomy at the Second Affiliated Hospital of Wenzhou Medical University between 2013 and 2018. HPV testing was performed with a nucleic acid genotyping kit for HPV (flow cytometry fluorescence hybridization method, Tellgen, Shanghai). After the detection of HPV within 1 month before the surgery, 20 HPV-negative and 20 HPV-positive cervical cancer tissues were collected and identified. Furthermore, another 20 normal cervical tissues were obtained from patients who underwent radical hysterectomy due to uterine adenomyosis, which was pathologically confirmed as normal cervical tissue or chronic cervicitis. Patients did not receive chemotherapy or radiotherapy before surgery. IHC was performed in cancer tissues using a previously described method.^45^ This study was approved by the ethics committee of the Second Affiliated Hospital of Wenzhou Medical University.

### Kaplan-Meier analysis

The Kaplan-Meier analysis was performed by the R package (https://cran.r-project.org/web/packages/survival/citation.html) to assess independent predictors of overall survival. Three groups were tested: 22 patients with HPV-negative cervical cancer; 281 patients with HPV-positive cervical cancer; and 304 patients with cervical cancer (one person did not have a determined HPV infection history). All survival information of cervical cancer patients was obtained from the TCGA database. The mRNA expression status separated the cases into “low” and “high” according to the comparison between expression values with median cutoffs values. The log rank test was applied to detect significant differences between groups.

### Systematical analysis

The expression levels of differentially expressed genes obtained from gene expression analysis section were validated by qRT-PCR for transcriptional levels and by western blotting for translational levels in HPV-negative cervical cancer cell lines. Moreover, IHC was used to measure the protein expression of these genes in HPV-positive cervical cancer tissues compared with HPV-negative cervical cancer tissues and normal control tissues (Table 3).

**Table 3:** Upregulated differentially expressed genes specifically in HPV-negative cervical cancer

Gene name	LogFC	LogCPM	p value	FDR
PRAME	7.3191	7.0601	0.0002	0.0113
HMGA2	6.2526	5.0822	0.0013	0.0483
MT1G	5.2074	4.5062	0.0009	0.0365
SBK1	4.5674	5.7858	0.0003	0.0143
ETV4	3.6619	6.1276	0.0005	0.0243
SLC7A5	3.5818	8.3032	0.0012	0.0464
MEX3A	3.5549	5.8684	0.0005	0.0232
PLS1	3.1988	5.5935	0.0010	0.0411
TM7SF2	2.8849	5.6680	0.0007	0.0313
RHPN1	2.8480	5.5456	0.0009	0.0377
PYCR1	2.5412	6.7111	0.0012	0.0463
SLC19A1	2.2630	5.0747	0.0002	0.0088
CHD7	2.1742	5.6171	0.0010	0.0410
TTYH3	2.0264	7.2369	0.0002	0.0101
HSPH1	1.7805	7.6452	0.0010	0.0402
SLC25A13	1.7218	5.6570	0.0007	0.0299
NIPSNAP1	1.7116	6.8293	0.0013	0.0492

FDR, false discovery rate; LogCPM, log counts per million, which can be understood as measuring expression level; LogFC, log fold change, which is the log difference between your groups

### Statistical analysis

Data were analyzed with GraphPad Prism 8.0 software. The significance of differences was determined using unpaired t tests with Welch’s correction for RT-PCR, western blotting, and IHC experiments. Data are presented as the means ± standard deviations. The log rank test was applied to detect significant differences between groups for Kaplan-Meier analysis. p <0.05 was considered statistically significant. It is important to note that less numbers of HPV-negative cervical cancer cases and only 3 normal cervical tissues of TCGA database may compromise the data in our study.

## References

[1] Ward Z.J., Grover S., Scott A.M., Woo S., Salama D.H., Jones E.C., El-Diasty T., Pieters B.R., Trimble E.L., Vargas H.A. (2020). The role and contribution of treatment and imaging modalities in global cervical cancer management: survival estimates from a simulation-based analysis. Lancet Oncol..

[2] Arbyn M., Weiderpass E., Bruni L., de Sanjosé S., Saraiya M., Ferlay J., Bray F. (2020). Estimates of incidence and mortality of cervical cancer in 2018: a worldwide analysis. Lancet Glob. Health.

[3] Siegel R.L., Miller K.D., Fuchs H.E., Jemal A. (2021). Cancer statistics, 2021. CA Cancer J. Clin..

[4] Crosbie E.J., Einstein M.H., Franceschi S., Kitchener H.C. (2013). Human papillomavirus and cervical cancer. Lancet.

[5] Hildesheim A., Gonzalez P., Kreimer A.R., Wacholder S., Schussler J., Rodriguez A.C., Porras C., Schiffman M., Sidawy M., Schiller J.T. (2016). Impact of human papillomavirus (HPV) 16 and 18 vaccination on prevalent infections and rates of cervical lesions after excisional treatment. Am. J. Obstet. Gynecol..

[6] Rodríguez-Carunchio L., Soveral I., Steenbergen R.D., Torné A., Martinez S., Fusté P., Pahisa J., Marimon L., Ordi J., del Pino M. (2015). HPV-negative carcinoma of the uterine cervix: a distinct type of cervical cancer with poor prognosis. BJOG.

[7] Katki H.A., Schiffman M., Castle P.E., Fetterman B., Poitras N.E., Lorey T., Cheung L.C., Raine-Bennett T., Gage J.C., Kinney W.K. (2013). Five-year risks of CIN 3+ and cervical cancer among women with HPV-positive and HPV-negative high-grade Pap results. J. Low. Genit. Tract Dis..

[8] Nicolas I., Marimon L., Barnadas E., Saco A., Rodriguez-Carunchio L., Fuste P., Marti C., Rodriguez-Trujillo A., Torne A., Del Pino M. (2019). HPV-negative tumors of the uterine cervix. Mod. Pathol..

[9] Liu D., Zhou P., Zhang L., Wu G., Zheng Y., He F. (2011). Differential expression of Oct4 in HPV-positive and HPV-negative cervical cancer cells is not regulated by DNA methyltransferase 3A. Tumour Biol..

[10] Kwasniewska A., Postawski K., Gozdzicka-Jozefiak A., Kwasniewski W., Grywalska E., Zdunek M., Korobowicz E. (2011). Estrogen and progesterone receptor expression in HPV-positive and HPV-negative cervical carcinomas. Oncol. Rep..

[11] Wang X., Wang G., Zhang L., Cong J., Hou J., Liu C. (2018). LncRNA PVT1 promotes the growth of HPV positive and negative cervical squamous cell carcinoma by inhibiting TGF-β1. Cancer Cell Int..

[12] Zheng Z., Gao Y. (2018). Down-regulation of lncRNA snaR is correlated with postoperative distant recurrence of HPV-negative cervical squamous cell carcinoma. Biosci. Rep..

[13] Ju W., Luo X., Zhang N. (2019). LncRNA NEF inhibits migration and invasion of HPV-negative cervical squamous cell carcinoma by inhibiting TGF-β pathway. Biosci. Rep..

[14] Liu Y., Li M., Yu H., Piao H. (2019). LncRNA SRA1 is down-regulated in HPV-negative cervical squamous cell carcinoma and regulates cancer cell behaviors. Biosci. Rep..

[15] Xu Y., Zou R., Wang J., Wang Z.W., Zhu X. (2020). The role of the cancer testis antigen PRAME in tumorigenesis and immunotherapy in human cancer. Cell Prolif..

[16] Al-Khadairi G., Decock J. (2019). Cancer testis antigens and immunotherapy: where do we stand in the targeting of PRAME?. Cancers (Basel).

[17] Ikeda H., Lethé B., Lehmann F., van Baren N., Baurain J.F., de Smet C., Chambost H., Vitale M., Moretta A., Boon T., Coulie P.G. (1997). Characterization of an antigen that is recognized on a melanoma showing partial HLA loss by CTL expressing an NK inhibitory receptor. Immunity.

[18] Tajeddine N., Gala J.L., Louis M., Van Schoor M., Tombal B., Gailly P. (2005). Tumor-associated antigen preferentially expressed antigen of melanoma (PRAME) induces caspase-independent cell death in vitro and reduces tumorigenicity in vivo. Cancer Res..

[19] Gattas G.J., Quade B.J., Nowak R.A., Morton C.C. (1999). HMGIC expression in human adult and fetal tissues and in uterine leiomyomata. Genes Chromosomes Cancer.

[20] Jiang Y., Zhu C., He D., Gao Q., Tian X., Ma X., Wu J., Das B.C., Severinov K., Hitzeroth I.I. (2019). Cytological immunostaining of HMGA2, LRP1B, and TP63 as potential biomarkers for triaging human papillomavirus-positive women. Transl. Oncol..

[21] Wang W.Y., Cao Y.X., Zhou X., Wei B., Zhan L., Fu L.T. (2018). HMGA2 gene silencing reduces epithelial-mesenchymal transition and lymph node metastasis in cervical cancer through inhibiting the ATR/Chk1 signaling pathway. Am. J. Transl. Res..

[22] Hu Z., Zhu D., Wang W., Li W., Jia W., Zeng X., Ding W., Yu L., Wang X., Wang L. (2015). Genome-wide profiling of HPV integration in cervical cancer identifies clustered genomic hot spots and a potential microhomology-mediated integration mechanism. Nat. Genet..

[23] Kenny S.L., McBride H.A., Jamison J., McCluggage W.G. (2012). Mesonephric adenocarcinomas of the uterine cervix and corpus: HPV-negative neoplasms that are commonly PAX8, CA125, and HMGA2 positive and that may be immunoreactive with TTF1 and hepatocyte nuclear factor 1-β. Am. J. Surg. Pathol..

[24] Günther K., Foraita R., Friemel J., Günther F., Bullerdiek J., Nimzyk R., Markowski D.N., Behrens T., Ahrens W. (2017). The stem cell factor HMGA2 is expressed in non-HPV-associated head and neck squamous cell carcinoma and predicts patient survival of distinct subsites. Cancer Epidemiol. Biomarkers Prev..

[25] Gallego M.I., Schoenmakers E.F., Van de Ven W.J., Lazo P.A. (1997). Complex genomic rearrangement within the 12q15 multiple aberration region induced by integrated human papillomavirus 18 in a cervical carcinoma cell line. Mol. Carcinog..

[26] Oh S., Shin S., Janknecht R. (2012). ETV1, 4 and 5: an oncogenic subfamily of ETS transcription factors. Biochim. Biophys. Acta.

[27] Zhang Y., Yokoyama S., Herriges J.C., Zhang Z., Young R.E., Verheyden J.M., Sun X. (2016). E3 ubiquitin ligase RFWD2 controls lung branching through protein-level regulation of ETV transcription factors. Proc. Natl. Acad. Sci. USA.

[28] Suriben R., Kaihara K.A., Paolino M., Reichelt M., Kummerfeld S.K., Modrusan Z., Dugger D.L., Newton K., Sagolla M., Webster J.D. (2015). β-cell insulin secretion requires the ubiquitin ligase COP1. Cell.

[29] Dumortier M., Ladam F., Damour I., Vacher S., Bièche I., Marchand N., de Launoit Y., Tulasne D., Chotteau-Lelièvre A. (2018). ETV4 transcription factor and MMP13 metalloprotease are interplaying actors of breast tumorigenesis. Breast Cancer Res..

[30] Kedage V., Selvaraj N., Nicholas T.R., Budka J.A., Plotnik J.P., Jerde T.J., Hollenhorst P.C. (2016). An interaction with Ewing’s sarcoma breakpoint protein EWS defines a specific oncogenic mechanism of ETS factors rearranged in prostate cancer. Cell Rep..

[31] Xiao J., Yang S., Shen P., Wang Y., Sun H., Ji F., Zhou D. (2017). Phosphorylation of ETV4 at Ser73 by ERK kinase could block ETV4 ubiquitination degradation in colorectal cancer. Biochem. Biophys. Res. Commun..

[32] Rodriguez A.C., Vahrenkamp J.M., Berrett K.C., Clark K.A., Guillen K.P., Scherer S.D., Yang C.H., Welm B.E., Janát-Amsbury M.M., Graves B.J., Gertz J. (2020). ETV4 is necessary for estrogen signaling and growth in endometrial cancer cells. Cancer Res..

[33] Xu L., Hu H., Zheng L.S., Wang M.Y., Mei Y., Peng L.X., Qiang Y.Y., Li C.Z., Meng D.F., Wang M.D. (2020). ETV4 is a theranostic target in clear cell renal cell carcinoma that promotes metastasis by activating the pro-metastatic gene FOSL1 in a PI3K-AKT dependent manner. Cancer Lett..

[34] Zwerger M., Kolb T., Richter K., Karakesisoglou I., Herrmann H. (2010). Induction of a massive endoplasmic reticulum and perinuclear space expansion by expression of lamin B receptor mutants and the related sterol reductases TM7SF2 and DHCR7. Mol. Biol. Cell.

[35] Bellezza I., Gatticchi L., del Sordo R., Peirce M.J., Sidoni A., Roberti R., Minelli A. (2015). The loss of Tm7sf gene accelerates skin papilloma formation in mice. Sci. Rep..

[36] Luteijn R.D., Zaver S.A., Gowen B.G., Wyman S.K., Garelis N.E., Onia L., McWhirter S.M., Katibah G.E., Corn J.E., Woodward J.J., Raulet D.H. (2019). SLC19A1 transports immunoreactive cyclic dinucleotides. Nature.

[37] Ritchie C., Cordova A.F., Hess G.T., Bassik M.C., Li L. (2019). SLC19A1 is an importer of the immunotransmitter cGAMP. Mol. Cell.

[38] Bufalieri F., Caimano M., Lospinoso Severini L., Basili I., Paglia F., Sampirisi L., Loricchio E., Petroni M., Canettieri G., Santoro A. (2020). The RNA-binding ubiquitin ligase MEX3A affects glioblastoma tumorigenesis by inducing ubiquitylation and degradation of RIG-I. Cancers (Basel).

[39] Wang X., Shan Y.Q., Tan Q.Q., Tan C.L., Zhang H., Liu J.H., Ke N.W., Chen Y.H., Liu X.B. (2020). MEX3A knockdown inhibits the development of pancreatic ductal adenocarcinoma. Cancer Cell Int..

[40] Yang D., Jiao Y., Li Y., Fang X. (2020). Clinical characteristics and prognostic value of MEX3A mRNA in liver cancer. PeerJ.

[41] Halleran A.D., Sehdev M., Rabe B.A., Huyck R.W., Williams C.C., Saha M.S. (2015). Characterization of tweety gene (ttyh1-3) expression in Xenopus laevis during embryonic development. Gene Expr. Patterns.

[42] Han Y.E., Kwon J., Won J., An H., Jang M.W., Woo J., Lee J.S., Park M.G., Yoon B.E., Lee S.E. (2019). Tweety-homolog (*Ttyh*) family encodes the pore-forming subunits of the swelling-dependent volume-regulated anion channel (VRAC_swell_) in the brain. Exp. Neurobiol..

[43] Weinberg F., Griffin R., Fröhlich M., Heining C., Braun S., Spohr C., Iconomou M., Hollek V., Röring M., Horak P. (2020). Identification and characterization of a BRAF fusion oncoprotein with retained autoinhibitory domains. Oncogene.

[44] Saha S.K., Biswas P.K., Gil M., Cho S.G. (2019). High expression of *TTYH3* is related to poor clinical outcomes in human gastric cancer. J. Clin. Med..

[45] Lu E., Hu X., Pan C., Chen J., Xu Y., Zhu X. (2020). Up-regulation of peroxiredoxin-1 promotes cell proliferation and metastasis and inhibits apoptosis in cervical cancer. J. Cancer.

